# Carbon fluxes resulting from land-use changes in the Tamaulipan thornscrub of northeastern Mexico

**DOI:** 10.1186/1750-0680-3-6

**Published:** 2008-09-30

**Authors:** Jose de Jesus Návar-Chaidez

**Affiliations:** 1 Professor, CIIDIR-IPN Unidad Durango. Sigma s/n. Fracc. 20 de Noviembre II. Durango, Dgo. Mexico. CP 34220

## Abstract

Information on carbon stock and flux resulting from land-use changes in subtropical, semi-arid ecosystems are important to understand global carbon flux, yet little data is available. In the Tamaulipan thornscrub forests of northeastern Mexico, biomass components of standing vegetation were estimated from 56 quadrats (200 m^2 ^each). Regional land-use changes and present forest cover, as well as estimates of soil organic carbon from chronosequences, were used to predict carbon stocks and fluxes in this ecosystem.

For the period of 1980–1996, the Tamaulipan thornscrub is presenting an annual deforestation rate of 2.27% indicating that approximately 600 km^2 ^of this plant community are lost every year and that 60% of the original Mexican Tamaulipan thornscrub vegetation has been lost since the 1950's. On the other hand, intensive agriculture, including introduced grasslands increased (4,000 km^2^) from 32 to 42% of the total studied area, largely at the expense of the Tamaulipan thornscrub forests. Land-use changes from Tamaulipan thornscrub forest to agriculture contribute 2.2 Tg to current annual carbon emissions and standing biomass averages 0.24 ± 0.06 Tg, root biomass averages 0.17 ± 0.03 Tg, and soil organic carbon averages 1.80 ± 0.27 Tg. Land-use changes from 1950 to 2000 accounted for Carbon emissions of the order of 180.1 Tg. Projected land-use changes will likely contribute to an additional carbon flux of 98.0 Tg by the year 2100. Practices to conserve sequester, and transfer carbon stocks in semi-arid ecosystems are discussed as a means to reduce carbon flux from deforestation practices.

## Background

Carbon dioxide concentrations in the atmosphere have increased by nearly 30% since the beginning of the Industrial Revolution [1-3]. The climatic and other environmental consequences of increasing carbon dioxide concentrations in the atmosphere have been widely discussed [4]. Carbon is released to the atmosphere by a variety of activities including the combustion of fossil fuels, deforestation, land-use changes, and cement production [5].

Deforestation and other land-use changes have contributed on the average 28% of the total global carbon dioxide emissions for the period between 1980 and 1998 [6]. Forest ecosystems cover more than 4.1 × 10^9 ^hectares of the Earth's land area [7] and contain about 1146 Pg C, with approximately 378 Pg in vegetation and the remaining 768 Pg in soils. Assessments of CO_2 _emissions from deforestation practices have been conducted at global and regional scales [e.g., [5,7-10]], at national scales [11,12], and at local levels [13-15]. Most studies at local or regional scales have focused on tropical regions, but little is known about carbon emissions of subtropical, semi-arid ecosystems. Masera et al., [11] suggested that conversion of forest lands to other uses can be contributing as much as 41% of the CO_2 _for Mexico and for the globe it is approximately 25% [5]. More realistic assessments must be conducted at local scales to improve understanding and to evaluate the feasibility of conducting practices leading to increasing carbon stocks to reduce this flux.

The aim of this study is to estimate the carbon flux associated with land-use change in the Tamaulipan thornscrub of northeastern Mexico as an example of the contribution to carbon flux of subtropical, semi-arid ecosystems. Management options for conserving stocks and sequestering carbon are discussed for the Tamaulipan thornscrub of northeastern Mexico with the possibility of extending these options to other areas.

## Results

### Estimated aboveground and root biomass components

Observed aboveground biomass components measured during the period of 1999–2002 are presented in Table 1. The spatial variation of biomass components was quite large, and most variation was observed between Mexican states. The semi-arid northwestern portion had average and confidence intervals (P = 95%) of biomass components of 12.93 ± 3.24 Mg ha^-1^, 10.17 ± 1.82 Mg ha^-1^, and 23.10 ± 5.06 Mg ha^-1 ^for aboveground, root, and total standing biomass, respectively. The other two locations had 48.40 ± 7.40 Mg ha^-1^, 30.04 ± 4.15 Mg ha^-1^, and 78.44 ± 11.54 Mg ha^-1 ^for aboveground, root, and total standing biomass, respectively. Total weighted root biomass estimates approximated 15.62 Mg ha^-1 ^and accounted for 64% and 39% of the total aboveground and total standing biomass, respectively. That is, weighted carbon stocks in biomass components of Tamaulipan thornscrub are 60% in aboveground biomass (11.35 Mg ha^-1^) and 40% in root biomass (7.81 Mg ha^-1^).

**Table 1:** Carbon estimates for the period 1999–2002 in plant compartments and soils in the Tamaulipan thornscrub ecosystem of three Mexican States located within northeastern Mexico.

Parameter	Mexican State			
		
	Coahuila	Nuevo Leon	Tamaulipas	
Weighted average by state area				

Carbon Density Parameter (Mg ha^-1^)				

Aboveground (TTF)	6.47	15.34	15.34	11.35*
Aboveground (Fallow)	3.00	7.11	7.11	5.25*
Roots (TTF)	5.09	10.06	10.06	7.81*
Roots (Fallow)	3.15	5.45	5.45	5.84*
Soils (TTF)	77.90	184.64	184.64	136.46*
Soils (Ag C)	32.65	77.38	77.38	57.19*
Soils (Fallow)	37.06	87.84	87.84	64.92*

TTF = Tamaulipan thornscrub forest, Fallow period for 15 years, Ag C = carbon content in agricultural soils cultivated for 15 years.

### Estimated aboveground and root carbon components in fallow lands

In fallow lands, carbon stocks in aboveground biomass reach a weighted average of 0.44, 2.11, and 5.25 Mg ha^-1 ^for sites with 5, 10, and 15 years of abandonment, respectively. The mean periodic and mean annual biomass increment is: 0.61 and 0.24; 1.36 and 0.57; and 2.21 and 0.95 Mg ha^-1 ^year^-1^, for sites with 5, 10, and 15 years of abandonment, respectively. That is, in 15 years of fallow, carbon stocks in aboveground biomass attain less than 50% of the carbon stocks in the original Tamaulipan thornscrub. Carbon stocks in root biomass attain a weighted average of 5.84 Mg ha^-1 ^in sites with 15 years of abandonment.

### Soil carbon stocks in the Tamaulipan thornscrub forest

The soil contains the largest C pool in the Tamaulipan thornscrub ecosystem. It has a weighted average of approximately 136.5 Mg ha^-1^, or 88% of the total organic carbon in the Tamaulipan thornscrub of northeastern Mexico. The rates of soil carbon accumulation during the establishment of Tamaulipan thornscrub in aboveground and root biomass and soils is approximately 5.25, 3.28, and 7.72 Mg ha^-1 ^in 15 years of site abandonment from agricultural practices. These values translated to annual rates per m^-2 ^approximates to 35, 22, and 51 g m^-2 ^y^-1 ^and they are consistent with values reported by Post and Kwon [16] for several semi-arid ecosystems.

### Land-use changes in the Tamaulipan thornscrub of northeastern Mexico

**Figure 1:**
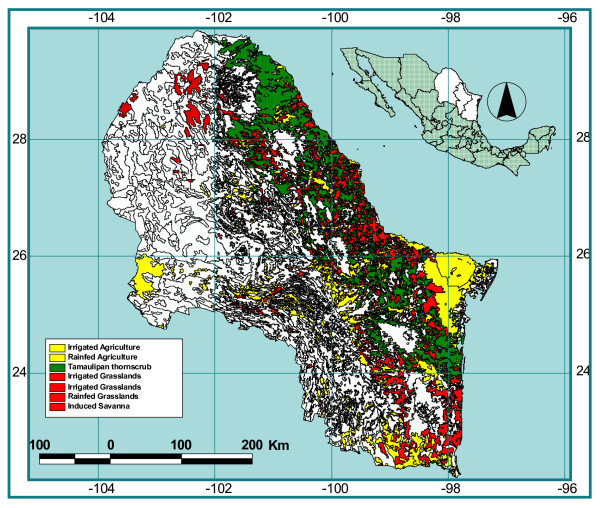
The distribution range of plant cover in northeastern Mexico, including Tamaulipan thornscrub, agriculture and grasslands.

The land-use changes from 1980 to 1996 observed in a 30,000 km^2 ^block placed in the center of the distribution of the Tamaulipan thornscrub of northeastern Mexico are large (Table 2). The disappearance of Tamaulipan thornscrub forest correlates to the appearance of agriculture and grasslands in the region (Figure 1). The proportion of the total studied area (30,000 km^2^) covered by native Tamaulipan thornscrub forests estimated from the 1980 data was 42% (12 282 km^2^) and only 27% remained intact for 1996 (4 905 km^2^). Intensive agriculture, including induced grasslands increased from 32 to 42% of the total studied area. This relationship implies that the significant increase in area converted to mainly agricultural fields (3,000 km^2^) and grasslands (1,000 km^2^) occurred largely at the expense of the Tamaulipan thornscrub forests. Plant communities dominated by *Prosopis *and *Acacia *forests and degraded forms of scrublands accounted for by the rest of the Tamaulipan thornscrub area lost during the studied period. Areas with gentle slopes, characterized by deep dark soils, are the most affected by shifting cultivation practices and intensive agriculture. Fallow lands, on the other side, increased at the expense of Mezquitales (1 000 km^2^) and abandoned agriculture and grasslands (1 500 km^2^). The annual rate of abandoned, fallow lands approximates to 2.33%, and surprisingly, this figure is quite similar to the annual deforestation rate. That is, for the study period, the area cleared is approximately the same as the area abandoned from other land uses which may eventually be converted to Tamaulipan thornscrub forests.

**Table 2:** Lost area and land-cover change of several plant communities in the Tamaulipan thornscrub ecosystem of northeastern, Mexico for the period of 1980–1996.

Year	Estimated Area (km^2^)	Area (km^2^)	Change (%)	Area Change per year (km^2^)	Annual Change rate (%)
Tamaulipan thornscrub					

1980	12,282.43	-4,462.38	-36.67	-278.89	-2.27
1996	7,820.05				

Agriculture					

1980	7,192.08	+2,637.84	+36.33	+164.86	+2.29
1996	9,829.92				

Grasslands					

1980	2,042.38	+541.02	+26.49	+33.81	+1.66
1996	2,583.40				

The annual rate of clearing Tamaulipan thornscrub is approximately 2.37%. That is, according to the last forest inventory, this ecosystem covers an approximate area of 32,188 km^2^, and it is disappearing in the natural state at an annual average rate of 600 km^2^. This figure is consistent with land use changes reported in other studies for the region (Table 3). SARH [17] reported deforestation of 3350 km^2 ^resulting from a combination of the opening of the irrigation districts 'Bajo Rio Bravo' (2400 km^2^), 'Bajo Rio San Juan' (800 km^2^), and 'Las Lajas' (150 km^2^), which occurred because of the construction of the International reservoirs 'La Amistad' and 'Falcon' and the Mexican reservoir 'El Azúcar' between the 1950's and the 1970's. The state government of Nuevo Leon reported deforestation figures of 1800 km^2 ^between 1980 and 1986 [18]. Maldonado [19] found that 11% of the plains of the Gulf of Mexico of the State of Nuevo Leon were deforested between 1976 and 1986. Treviño et al., [20] reported for the municipality of Linares annual deforestation rates of 2.2% for the period of 1973 to 1994.

**Table 3:** Land-use changes from the 1950's to the 2000's in the Tamaulipan thornscrub of northeastern Mexico and southeastern USA.

Source	Period	Area (km^2^)		Rate (%)	Places
		Total	Deforested		

SARH (1980)	1950–1970	Not Available	3,400		Tamaulipas and Nuevo Leon
Proderleon (1990)	1980–1986		1,800		Nuevo Leon
Maldonado (1992)	1976–1986			1.10	Eastern Nuevo Leon
Treviño et al. (1996)	1973–1994	2,259	1,088	2.19	The Municipality of Linares, N.L.
Udvardy (1975)	1975	200,000			Mexico & US
WWF (2001)	2001	141,500	58,500	1.17	Mexico & US
WWF (2001)	2020	106,125	35,375	1.25	Mexico & US
Estimates	1975	45,495			Mexico
Palacios- Prieto (2000)	2001	32,188	13,307	1.17	Mexico
Predicted	2020	24,141	8,047	1.25	Mexico

Considering the average annual deforestation rate (1.85%) estimated from a) this study as 2.37%, b) by Treviño et al., [20] for the period of 1973–1994 as 2.2%, and c) by Maldonado [19] for the period of 1976–1986 as 1.1%, and current area reported by Palacios-Prieto [21], the Tamaulipan thornscrub of northeastern Mexico would have covered an area of 80,490 km^2 ^in 1950. This estimate compares to the deforestation area reported by the state of Nuevo Leon (1800 km^2^) for the period of 1980–1986 [18] and the sum all 6-year periods since 1950 and for the other two states (Tamaulipas and Coahuila). Considering the average rate of deforestation of 1.85%, by the year 2000 there was 60% less area covered by Tamaulipan thornscrub in northeastern Mexico. The World Wildlife Fund [22] estimated that over 25% (8 047 km2) of this ecoregion will disappear due to changes in land-use by the year 2020 since this rate will reduce the area covered by Tamaulipan thornscrub by 31% of the area reported by Palacios-Prieto [21]. Therefore, under this deforestation scenario, the area covered by this ecosystem would be 28% (22 156 km^2^) and 6% (4 974 km^2^) of the area covered in 1950 for the years 2020 and 2100, respectively.

### Carbon fluxes by land-use changes

**Figure 2:**
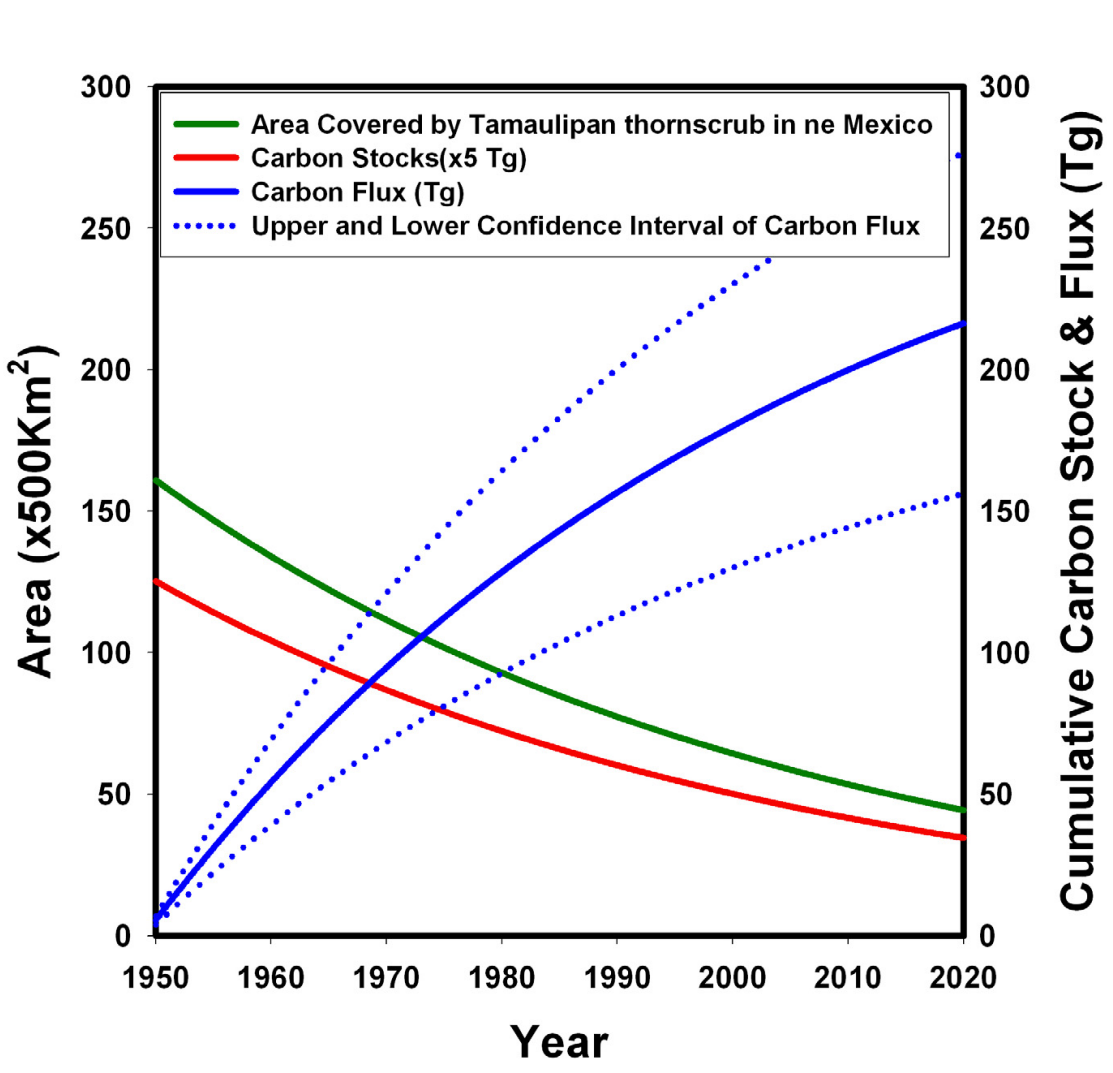
Land-use change and carbon flux resulting from land-use change in the Tamaulipan thornscrub of northeastern Mexico.

The carbon released in this ecosystem due to land-use changes was 180.1 Tg for the period between 1950–2000. Under the rate of the deforestation scenario, carbon fluxes for the period of 2000–2100 would be 98.0 Tg (Figure 2). For the year 2000, in general, clearing Tamaulipan thornscrub for agriculture is releasing carbon at an annual rate of 2.19 Tg C y^-1^ of which aboveground biomass and root biomass accounted for by 10 and 8% and soils explained the rest 88%. Shifting land use from agriculture to Tamaulipan thornscrub increases carbon stocks at an annual rate of 0.74 Tg C y^-1 ^of which aboveground and root biomass explained 16 and 9% and soil the remaining 76%. Therefore, there is a net release of carbon stock of 2.19 Tg C y^-1 ^of which soil looses approximately 1.80, aboveground biomass 0.22 and roots 0.17 Tg C y^-1^. The conversion of native forests and grasslands to farms has released globally about 100 Pg of carbon over the past 150 years [23]. During the 150 years scenario studied in this report (1950–2100), this ecosystem would likely release 0.278 Pg C.

## Conclusion

### Estimated aboveground and root biomass components

Biomass estimates are consistent with other biomass measurements and estimates in the Tamaulipan thornscrub of south central Nuevo Leon. Návar et al. [24] measured for the subtropical Tamaulipan thornscrub from 25 m^2 ^ha plots an average aboveground biomass of 44.4 Mg ha^-1^. Heiseke [25] reported biomass stock figures between 34.21 and 62.70 Mg ha^-1 ^in the plains and between 26.06 and 37.70 Mg ha^-1 ^in the hills and concluded that this biome has a maximum standing biomass of 50 Mg ha^-1^. Carstens [26] measured in this plant community between 35 and 47 Mg ha^-1^. Cairns et al. [14] recorded slightly smaller biomass figures for chaparrales and xerophytic vegetation of southern Mexico (24 and 28 Mg ha^-1^, respectively). Castellanos et al. [27] reported larger biomass figures for dry tropical forests of western Mexico (73 Mg ha^-1^). Root biomass estimates are consistent with estimates in cloud forest ecosystems, but they are smaller than reported figures for tropical ecosystems [15,27].

Total soil carbon stock per hectare in this ecosystem (138 Mg ha^-1^) is within the range of measurements and estimates reported by De Jong et al., [15] for secondary shrubs (average of 140 Mg ha^-1^), for pastures (120 Mg ha^-1^), and for cultivated lands (140 Mg ha^-1^) of the selva Lacandona in Chiapas Mexico. The Tamaulipan thornscrub has most of its carbon pool allocated in organic soil matter and aboveground biomass (weighted average of 136.46 Mg ha^-1 ^and 11.35 Mg ha^-1^, respectively). The ratio of standing biomass carbon/soil organic carbon approximates to 7% and it is consistent with the ratio reported by Watson et al. [6] for tropical savannas. De Jong et al., [13] summarized several studies on tropical ecosystems and observed that on the average the soil and standing biomass compartments are balanced with 120 Mg ha^-1^. Watson et al., [6] also reported similar carbon stocks in soils and standing biomass for tropical forests.

### Rates of deforestation in the Tamaulipan thornscrub ecosystem

Deforestation figures reported for the Tamaulipan thornscrub are also within the average recorded for several studies. For Mexico, for the period of 1993–2000, Semarnat [28] recorded deforestation rates of 3.93%. For the tropical rainforest of 'Los Tuxtlas' for the period of 1967–1986, Dirzo and Garcia [29] reported 4.3%. Cairns et al. [30], Cairns et al., [14], De Jong et al., [15], and Ochoa-Gaona [31] recorded similar deforestation figures for tropical forests of southern Mexico. Dirzo and García [29] also reported similar deforestation rates for tropical dry forests of western Mexico.

### Land-management options to conserve carbon sequestration in the Tamaulipan thornscrub ecosystem

This study suggests that eliminating land-use changes in the Tamaulipan thornscrub would conserve carbon stocks by 2.19 Tg annually. In addition, this action would provide other environmental benefits such as habitat conservation for diversity [22], soil and water conservation [32], buffering local microclimate and global climatic changes, and other additional benefits.

Deforestation promotes soil carbon emissions of the order of 1.8 Tg yr^-1 ^(82% of total emissions). Removals of standing aboveground and root biomass account for the remaining 18%. Conservation of SOC must be the primary concern and can be approached in several ways: (1) conserving the natural ecosystem, (2) protecting the soil carbon, and (3) sequestering soil carbon that has been lost.

The conservation of the natural ecosystem is of critical local, national, and global importance. The promotion of new policies on land change and management can reduce carbon emissions in the agricultural sector. For example, modifying the forestry law by reducing the scale of land-use change or increasing the time allotted for abandoned lands to recover the original plant community would cause less private and communal lands to be cleared. Conservation of carbon stocks under the new policy scenario is a function of the area allowed to be cleared and the time to return to forests. For example, at the present time, if fallow last 5 or 10 years, SOC sequestration would be reduced to 0.07 and 0.01 Tg C y^-1^, respectively. On the other hand, if fallow lasts for 25 years, SOC sequestration would be 0.52 Tg C y^-1^.

In recently cleared lands, conservation of the SOC must be the primary concern because organic carbon in total aboveground biomass would only be released, accounting for 10% of the total organic carbon stocks in the system. Currently, approximately 600 km^2 ^year^-1 ^are cleared for farming in this ecosystem and lose much soil organic carbon (65%) in 5 years [33]. Since most of the newly cleared lands are used for rain-fed agriculture, a feasible way to preserve carbon is to implement non-till, minimum tillage or tillage conservation practices at the beginning of farming. Of the area cleared each year, approximately 20 Tg C could be conserved in 10 years of no-till conservation practices. However, the expense of high fertilizer and pesticide expenditures used to control weeds may limit the ability of farmers to carry out these practices unless a carbon credit or payment to farmers is associated with the carbon sink in cropland agriculture.

Carbon can be sequestered in soils that have been under agricultural use for more than 15 years by promoting no-till or conservation practices. No-till and conservation tillage practices associated to efficient irrigation, fertilizer, and pesticide applications can increase SOC [34-38]. The increase can occur via several means: improving yields and subsequent organic matter additions to the soil or reductions in the rate of soil organic carbon loss [39], decreasing carbon dioxide emissions caused by the reduction of fossil-fuel use by tractors and other tillage equipment, and reducing the area cleared by increasing crop yield. Since the rate of carbon sequestration can be doubled in soil with depleted organic carbon [40], conservation tillage practices can be effectively carried out in the irrigation districts 'Bajo Bravo', 'Bajo San Juan', and 'Las Lajas', where agriculture is highly mechanized, irrigated, and with high inputs of fertilizers, pesticides, and herbicides. There are approximately 3500 km^2 ^available for this purpose within the distribution range of the Tamaulipan thornscrub in northeastern Mexico. The rate of carbon sequestration and an economic analysis of carbon flux in the irrigation districts require further study. The former is a function of the observed turnover time and the amount of active carbon in the system [40]. Irrigation schedules must be planned properly since leaching of humic substances can be a factor of reducing carbon sequestration in soils.

The potential of carbon sequestration by shifting from conventional tillage to no-tillage in agricultural soils is dependent on several factors [41]. In the US, carbon can be sequestered on average in the range of 337 ± 108 Kg C ha^-1 ^yr^-1 ^to a depth of 30 cm., and these rates vary from 300–600 kg C ha^-1 ^yr^-1 ^in the Great Plains of US, to 100–500 kg C ha^-1 ^yr^-1 ^in the Canadian prairie region [37]. This rate of carbon sequestration can continue for 20 years, and then it will decline [39]. Therefore, conversion from conventional tillage to no-till farming of the 3,500 km^2 ^of the irrigation districts could potentially sequester 2.4 Tg C in 20 years. Information on C sequestration by tillage operations is scarce in northeastern Mexico, however, our data on abandoned agricultural lands suggest that C sequestration in fallow lands could be on the order of 1000 kg ha^-1^ yr^-1^ when organic carbon in roots and above ground biomass is included in the estimate.

Economic incentives to promote carbon-conservation and sequestration practices in native forests and soils are not presently recognized by the United Nations Framework Convention on Climate Change. However, carbon sequestration in agricultural ecosystems soils may later be added to the Kyoto Protocol or other protocols that may develop as a response to climate change, and then carbon credits or payments coming from international funds would be available to farmers. In the meantime, Mexican forestry law is promoting local markets for the environmental benefits provided by forest ecosystems.

The deforestation is a cause of concern since world wide it is close to 13 M ha. Gurney and Raymond [42] proposed the approach called the 'Preservation Pathway' that combines the desire for forest preservation with the need to reduce emissions associated with forest loss by focusing on the relative rate of change of forest cover as a criteria by which countries gain access to trading preserved forest carbon stocks. To reduce deforestation in poor countries, Kindermann et al., [43] advanced a combination of incentives and taxes as financial mechanisms. The Preservation Pathway with a combination of incentives and taxes can be downscaled at the ecosystem level, since tropical countries and tropical ecosystems show the largest potential for carbon sequestration despite projections that most of the agricultural expansion will be in these regions [44].

## Conclusion

Semi-arid and subtropical semi-arid ecosystems comprise 28% of the globe [45]. Land-use changes in the Tamaulipan thornscrub ecosystem of northeastern Mexico have instigating changes in carbon stocks. Carbon stocks were observed primarily in soils (82%), standing aboveground biomass (10%), and roots (8%). Currently land-use changes have contributed to approximately 2.19 Tg C yr^-1 ^emissions by clearing approximately 300 km^2 ^yr^-1 ^and abandoning approximately 300 km yr^-1 ^to fallow lands. Several practices are discussed and recommended to conserve and sequester carbon in soils. This information is critical for sustainable management and understanding of several of the environmental services provided by the Tamaulipan thornscrub of northeastern Mexico. This type of analysis would be useful to conduct in other regions of subtropical, semi-arid ecosystems when information on carbon flux and land-use change are poor and where there is opportunity for improved carbon storage by means of changes in land-management practices.

## Methods

### The Tamaulipan thornscrub forest of northeastern Mexico

The Tamaulipan thornscrub forest is a subtropical, semi-arid ecosystem that distributes in the Mexican states of Coahuila, Nuevo Leon, and Tamaulipas, covering a total area of 32,188 km^2 ^according to the 2000 forest inventory by Palacios-Prieto [21]. See Figure 1. This ecosystem is limited to the northwest by the Chihuahuan Desert, to the west by the Sierra Madre Oriental mountain range, and to the south by the tropical rainforest of the Sierra Azul mountain range [46]. Native thornscrub forests historically dominate land cover in this region. However, shifting cultivation is a typical practice in the community-based land tenure system, *ejido*, which is rapidly reducing the area of the thornscrub ecosystem. Like other subtropical thornscrub forests in the world, the ecosystem has been extensively used as pastureland for the last 350 years [47] and, currently, is used for fuel, timber for fencing and construction, food, and medicinal plants [48,49]. Even though selective harvesting is common, it is lightly carried out and does not have a noticeable impact on the Tamaulipan thornscrub structure. Grazing is the major disturbance that promotes important structural changes in pasture lands [50,51].

In northeastern Mexico, the Tamaulipan thornscrub is a dense and diverse ecosystem. It contains at least four endemic genera of woody plants, which indicate a significantly high diversity of woody plant species [52]. It is also rich in terms of native cacti, as well as endangered plant species [53]. Spiny shrubs and low trees dominate the thornscrub, but grasses, forbs, and succulents are also prominent [54]. Romero [55] and Manzano and Návar [50] recorded 22 shrub species in 0.1 ha plots and more than 5000 shrubs per ha in 0.025 ha plots. Medium and small shrubs (less than 10 m in height) are common life forms, and they are disappearing because of selective harvesting for fuel wood and timber extraction and a reduction in the cycle of shifting cultivation practices in the area.

The southern part of the ecosystem is characterized by a moist, subtropical climate typical of southeastern Nuevo Leon and western Tamaulipas, while the northern bordering region is characterized by a semi-arid climate. Average annual precipitation ranges from 400 to 500 mm in the northern part of the three-bordering Mexican states; to 700 to 800 mm at the south central region, to 1000 mm at the piedmont of the Sierra Madre Oriental mountain range. Pan evaporation is less variable than annual precipitation and approximates 2000 mm in the plains of the northern Gulf of Mexico.

Litosols and rendzins dominate the hilly slopes at the piedmont of the Sierra Madre Oriental mountain range and the smaller isolated mesetas of the plains of the northern Gulf of Mexico. Yermosols and xerosols are widely distributed in the semi-arid western and northwestern region, and deep Vertisols dominate the lowlands of the plains of the northern Gulf of Mexico.

### Carbon stocks and fluxes

Carbon stocks in the Tamaulipan thornscrub ecosystem were estimated from biomass components in (a) aboveground vegetation, (b) roots, and (c) soils. The soil carbon of the litter layer was not included in the measurements since late field observations indicate that it may account for less 1 Mg ha^-1 ^and have large spatial variation.

Within the Tamaulipan thornscrub of northeastern Mexico, 56 plots (each 5 m × 40 m) were used to estimate aboveground biomass to derive carbon stocks in three locations. Quadrats were systematically placed following the distribution of the thornscrub from the northwestern to the southeastern portion: (a) the northwestern (NW) portion, covering the northern part of the states of Coahuila, Nuevo Leon, and Tamaulipas (21 plots), (b) the south-central (SC) region of Nuevo Leon (20 plots), and (c) the piedmont of the eastern Sierra Madre (SM) mountain range in the state of Nuevo Leon and Tamaulipas (15 plots). At each location, quadrats were stratified randomly placed to account for typical conditions and major sources of variation in the physical characteristics of the environment. All woody shrubs were measured for basal diameter (d), top height (h), and vertical projection of canopy cover (ct) by species (s). These data provided information to estimate aboveground biomass by applying 17 allometric equations derived by Návar et al. [56] to estimate total tree biomass.

There is a smooth transition of Tamaulipan thornscrub from subtropical to semiarid climates. Therefore, the area covered by this ecosystem in the semi-arid portion of Nuevo Leon and Tamaulipas is not well known. Therefore, average biomass densities for quadrats measured in the semi-arid (NW) and subtropical (SC and SM) plots were utilized to estimate biomass for these states. The NW portion is characterized by *P. glandulosa*, the SC by *D. texana*, *P. pallens*, and *A. berlandieri*, and the SM by *C. boissierii*, *B. myricaefolia*, and *P. pallens *as the most important plant species. This approach assumes that 50% of the area is dominated by *P. glandulosa *(semi-arid Tamaulipan thornscrub) and the remaining 50% by *D. texana*, *C. boissierii *and *P. pallens *(subtropical Tamaulipan thornscrub).

An independent equation derived by Návar et al., [57] was used to estimate biomass of roots with diameters larger than 1 mm. The equation uses total aboveground biomass as an independent variable and estimates root biomass in the A and B soil horizons. The same weighting procedure to calculate biomass density for each of the Mexican states was used to estimate root biomass.

Data on soil organic carbon (SOC) for the SC and SM locations were provided by Bravo [33] for undisturbed thornscrub forests, abandoned fallow lands, and for agricultural soils cultivated for 15 years. Undisturbed thornscrub forests and agriculture soils have approximately 43 and 15 g kg^-1^, respectively, in the top soil horizon, and SOC decays hyperbolically as a function of time and soil depth [58] as has been observed by Post et al., [59] in other parts of the world. The depth of tillage operations in the lowland plains of northern Mexico rarely exceeds 50 cm. Therefore, computations of SOC stocks and in our releases during cultivation and gains in fallow lands considered this soil depth as the most dynamic SOC stock in the soil system. Estimates of soil mass in the soil profile (50 cm of soil depth) were calculated according to:

M = Pb*V,

Where: M = soil mass (Mg ha^-1^), Pb = soil bulk density (Mg m^-3^); V = soil volume (m^3 ^ha^-1^). These equations were empirically derived from data reported by Bravo [58] for SOC, which are consistent with the mathematical functions reported by Post et al., [59] and for soil bulk density from Návar et al., [60]. The equations are:

$$\begin{array}{l} SOC(t)=14.193+\frac{26.60*1.81}{1.81*t}\\ Pb(z)=0.9223Z^{0.063}\\ SOC(z)=40.80*Exp^{-0.0089Z} \end{array}$$

Where: t = time (years), Z = soil depth (cm). Note that 40.80 g kg^-1 ^is the initial SOC content at the top soil surface for the Tamaulipan thornscrub. When Tamaulipan thornscrub forests are cleared, 40.80 g kg^-1 ^decays hyperbolically as a function of time and exponentially as a function of soil depth.

Changes in SOC recorded during fallow are also available for these computations. The rate of increase is linear, as reported by Bravo [58]:

*SOC*(*t*) = 14.5 + 0.3277* *fallow*·*length*

The rate of carbon increase, in aboveground biomass, *AC*(*t*), and root biomass, *RC*(*t*), were derived from choronosequence biomass data collected in abandoned fallow lands. The equation used to estimate root biomass from aboveground biomass was reported previously by Návar et al. [61]. Biomass growth reported from chronosequences is consistent with periodic and mean annual biomass increments recorded by Návar et al., [24]:

*AC*(*t*) = 0.0164*t*^2.2422^

*RC*(*t*) = 1.465 + 0.56 * [0.0164*t*^2.2422^]

The power equation to estimate aboveground biomass growth works well for fallow lands with less than 20 years of abandonment. Data from fallow lands with longer periods of time since abandonment is required to fit the classical s-shape function to biomass growth as a function of time.

Soil carbon data for the semi-arid NW region was not available (SOCnw); hence, it was calculated by multiplying the average SOC for the SC and SM (SOCsc and SOCsm) by the ratio of average total aboveground biomass for the NW (TAGBnw) and the average aboveground biomass for SC and SM (TAGBsc and TAGBsm locations). That is, (SOCnw/SOCsc, sm) = (TAGBnw/TAGBsc, sm). The justification for this equation is that plant cover controls the soil organic carbon content.

### Land-use changes

The spatial changes in plant cover through time were measured using Landsat MSS images from February 1980 and March 1996. These data were geometrically corrected, and an unsupervised classification was used to define spectral classes based on a maximum -likelihood clustering algorithm. Ten different spectral classes were obtained, identified, and confirmed during field work. Vegetation maps for 1980 and 1996 were overlaid in order to estimate the fate of land cover with special emphasis on Tamaulipan thornscrub ecosystem, agricultural uses, and grasslands.

Additional data on land-use changes were obtained from several sources [17-20] for different periods and locations encompassing the Tamaulipan thornscrub of northeastern Mexico. An average deforestation rate was estimated for the area covered by Tamaulipan thornscrub using land-use changes observed in this study and reported land-use changes, consistent with the total area covered by Tamaulipan thornscrub in Mexico and US for the 1950's [62,63] and 2000 [31]. An average deforestation rate has the advantage that it is consistent with other regional land-use change estimates and smoothes the short spatial and temporal changes, but it has the disadvantage in that it is fixed over time, since land-cover change is a disjointed process, with irregular periods of rapid change [64].

### Procedure for estimating carbon stocks and fluxes

The procedure for estimating carbon stocks and fluxes employed five steps: 1) an estimation was made of the average annual deforestation rate; 2) projections of the area covered by Tamaulipan thornscrub was done using the average annual deforestation rate and as a baseline the area recorded from the last forest inventory of 2000 [21]; 3) the rate of abandoned, fallow lands was estimated from other plant communities (grasslands, agriculture, scrublands) shifting to Tamaulipan thornscrub as a function of the area covered by Tamaulipan thornscrub and subtracted from the projected area covered by Tamaulipan thornscrub; 4) carbon stocks in aboveground and root biomass were derived from biomass estimates using allometric equations and employing a biomass to carbon content factor of 0.50; and 5) SOC mass estimates were based on projections of soil bulk density with soil depth, soil mass per hectare basis, the annual rate of SOC depletion by shifting cultivation using the hyperbolic equation, the annual SOC increment in abandoned, fallow, agricultural lands.

The major assumptions of this methodology are: a) the annual rate of deforestation and abandoned, fallow lands is constant over time, b) there is a continuous shifting cultivation cycle of clearing, agricultural use, and fallow lasting 15 years; c) there is a mosaic of farms with different times of agricultural use and fallow; d) in agricultural lands SOC depletes in time to a minimum over 15 years as specified by equation [2] and in fallow lands SOC recovers in 15 years according to equation [5]; aboveground and root biomass recover during fallow according to equations [6] and [7], respectively; e) the carbon content in biomass is 0.5; d) the soil bulk density function with soil depth remains constant in Tamaulipan thornscrub, agriculture, and fallow lands. This methodology is quite similar to other procedures widely used to estimate carbon stock and fluxes [13-15,29].

## Competing interests

The author declares that he has no competing interests.

## Authors' contributions

JN contributed entirely to this manuscript.
